# Reversed L-shaped incision for resection of a large azygos vein aneurysm: a case report

**DOI:** 10.1186/s40792-023-01669-w

**Published:** 2023-05-18

**Authors:** Kazuki Tamura, Yasunobu Konishi, Wataru Tatsuishi, Tomonobu Abe

**Affiliations:** Department of Cardiovascular Surgery, Gunma University Hospital, 3-39-15 Showamachi, Maebashi, Gunma 371-8511 Japan

**Keywords:** Azygos vein aneurysm, Reversed L incision, Thoracic surgery

## Abstract

**Background:**

Azygos vein aneurysms are rare and asymptomatic in many cases. The management for these aneurysms is controversial, and there is no clear guideline or evidence-based threshold for surgical or interventional therapy.

**Case presentation:**

Herein, we report the case of a giant azygos vein aneurysm in a 78-year-old man that was treated with a reversed L-shaped incision. A 56 × 77 mm saccular azygos vein aneurysm was incidentally detected on computed tomography. Subsequently, surgical resection with interventional radiology and reversed L-shaped thoracotomy was performed. First, we performed coil embolization of the azygos vein aneurysm inflow. Next, a cardiopulmonary bypass was established through a reversed L-shaped sternotomy, and the aneurysm was excised.

**Conclusions:**

In this case, surgical resection via reversed L incision was effective.

## Background

The majority of documented patients with an azygos vein aneurysm (AVA) was diagnosed incidentally by computed tomography (CT) or magnetic resonance imaging (MRI) as a mediastinal mass [1–5]. Large AVAs are associated with risks of rupture, pulmonary embolism, and thrombus formation. An open thoracotomy may be safer for cases of a large AVA with the risk of massive haemorrhage and intraoperative thrombus migration [5]. Herein, we present a case of surgical resection combined with reversed L-shaped thoracotomy, and interventional radiology (IVR).

## Case report

**Fig. 1:**
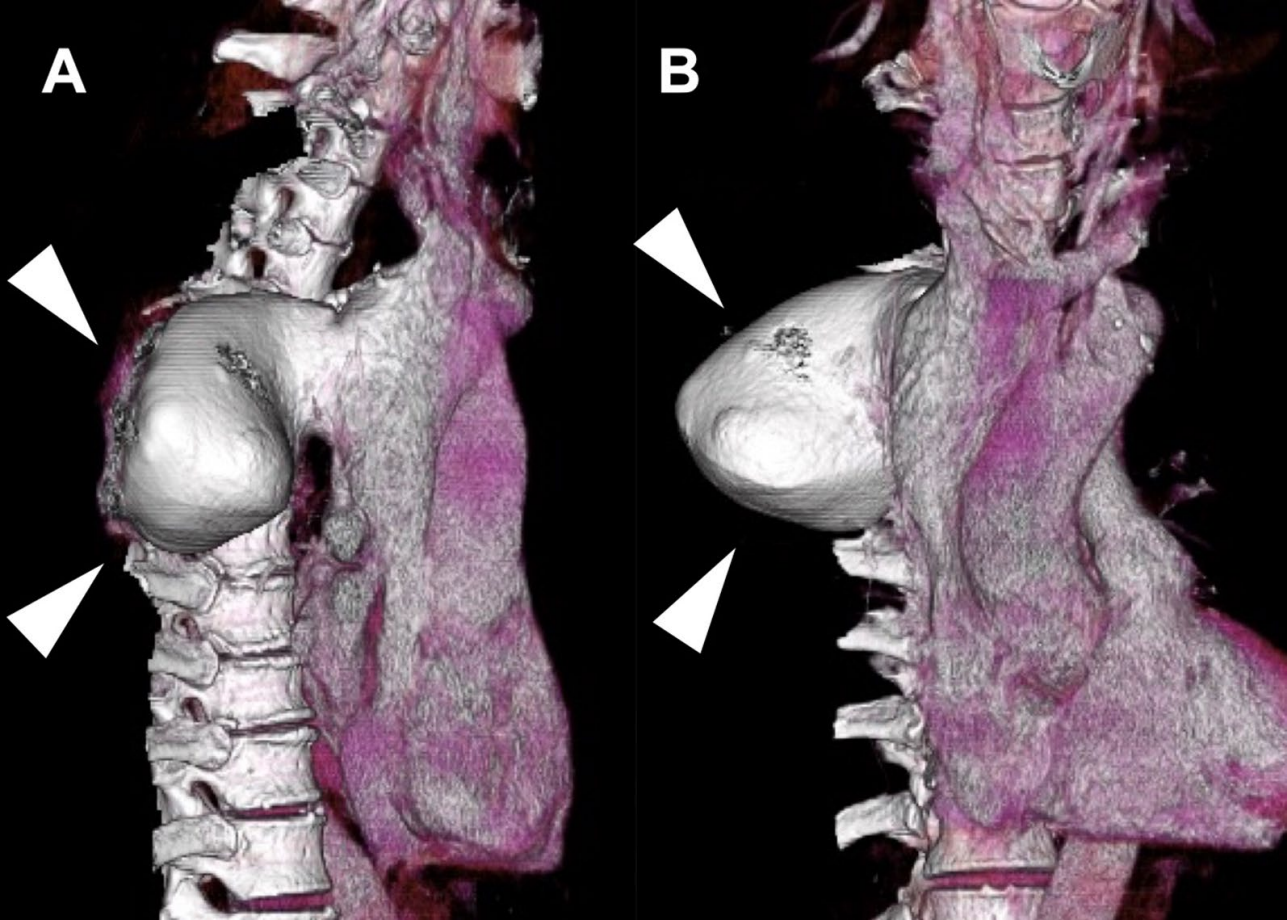
Three-dimensional reconstruction of a large AVA indicated by arrows (**A** lateral view, **B** front view)

**Fig. 2:**
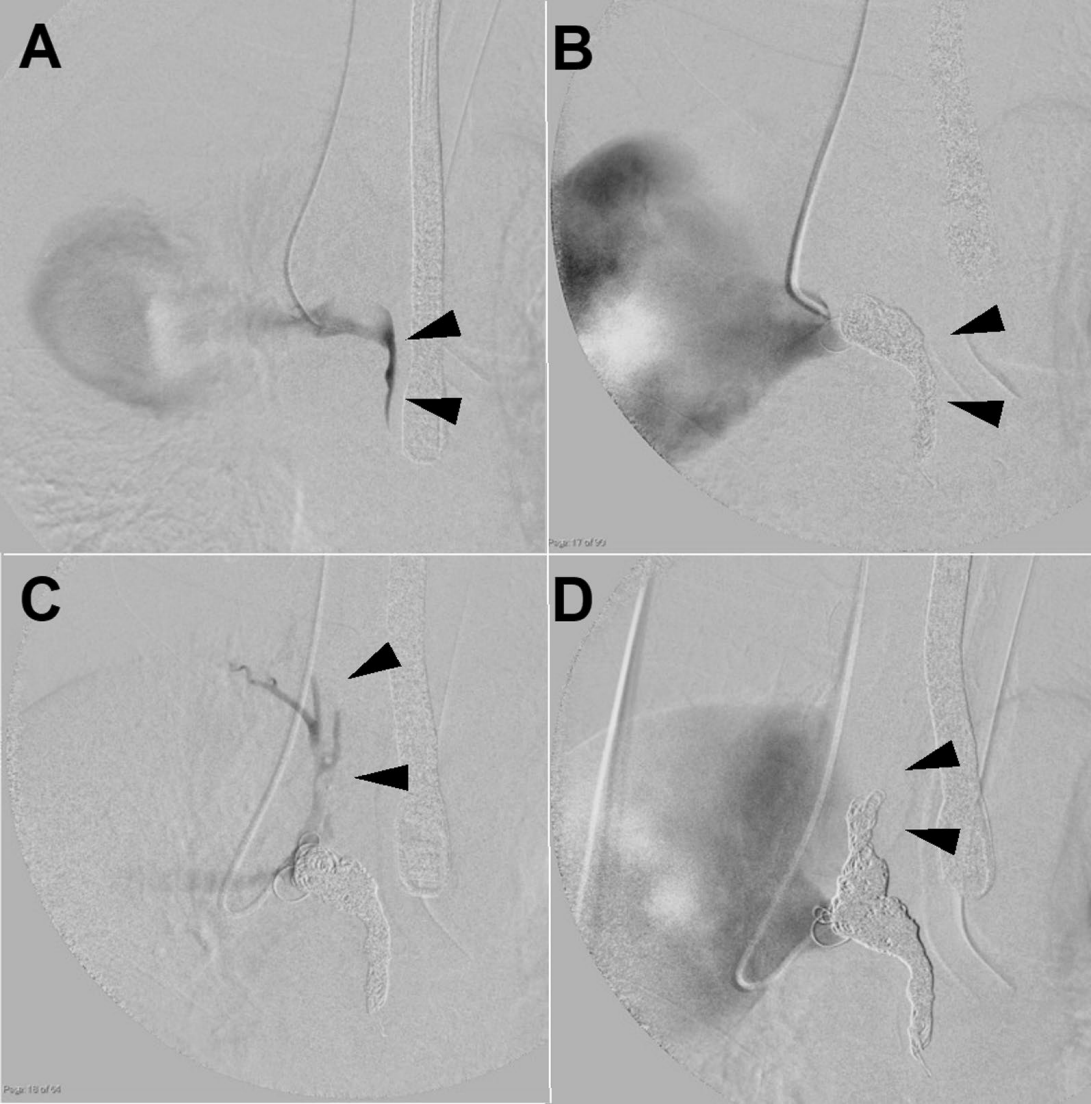
Intraoperative angiography. **A** It shows the AVA and caudal inflow(arrowheads). **B** After coil embolization of distal azygos vein (arrowheads). **C** It shows 2nd, 3rd, and 4th intercostal vein(arrowheads). **D** After coil embolization of intercostal veins (arrowheads)

**Fig. 3:**
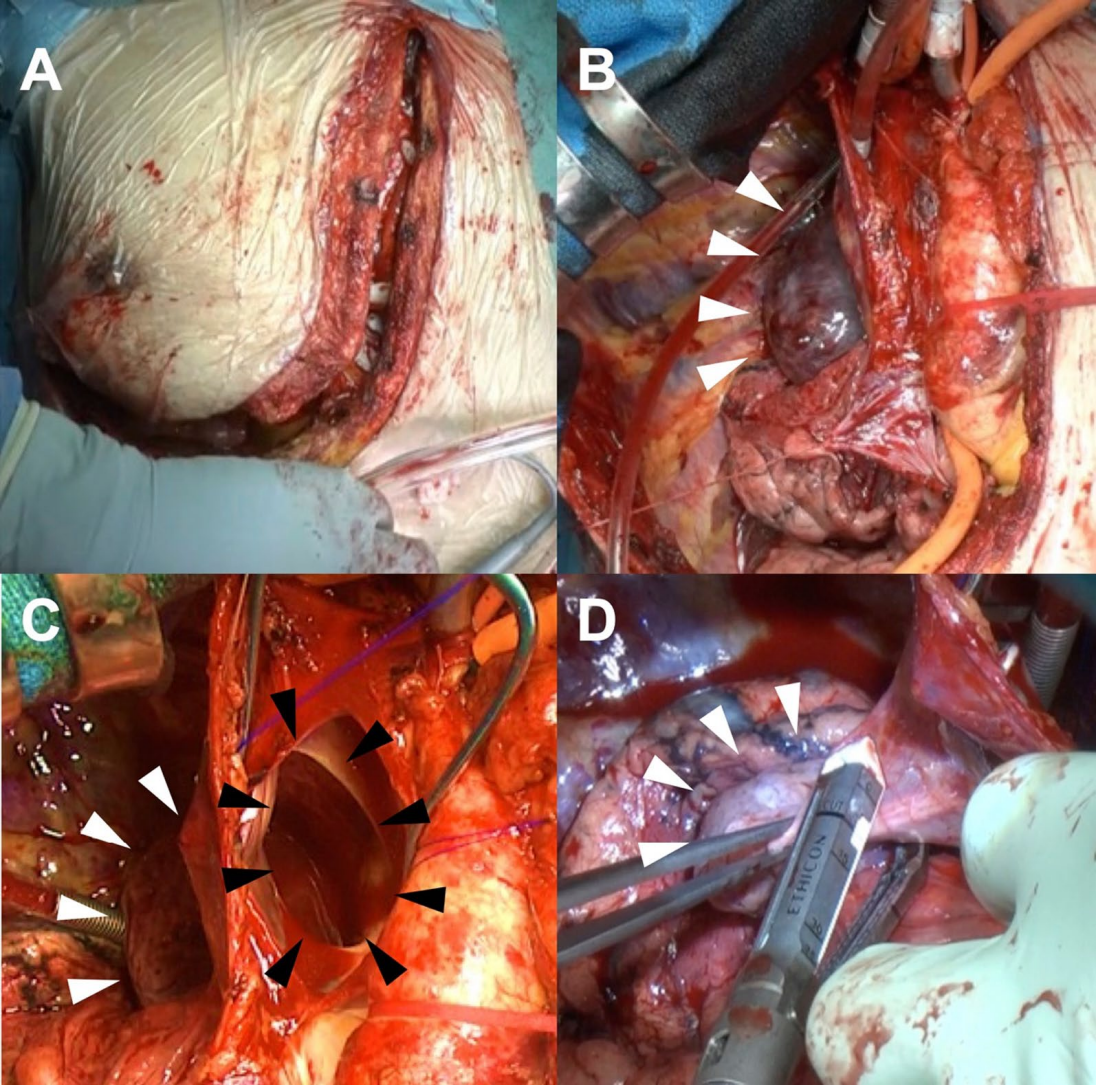
**A** Reversed L-shaped sternotomy. **B** Depiction of AVA (arrowheads). **C** After incising the SVC, the orifice of the azygos vein (black arrowheads) and depressurized AVA (white arrowheads) are revealed. **D** AVA excision using a vascular stapler (arrowheads)

A 78-year-old asymptomatic male patient underwent evaluation for prostate cancer. CT revealed a 56 × 77 mm mass at the posterior mediastinum (Fig. 1); subsequently, he was diagnosed with AVA. Apart from prostate cancer, his medical history was unremarkable. He did not have history of liver disfunction nor trauma.. In addition, the physical examination results and laboratory data were normal. When we had extensive discussion with the general thoracic surgeons, there was concern that there could be a strong adhesion to the surrounding tissues.The orifice of the azygos vein was larger than in previous cases reported as well. The chest surgeons suggested that it would be safer to perform the surgery under the cardiopulmonary bypass by cardiac surgeons considering the risk of major bleeding. We anticipated from the preoperative images that resection of the AVA would be difficult. Therefore, we planned to occlude the AVA inflow with coil embolization, and to surgically close the outflow from inside of the superior vena cava (SVC), thus excluding the aneurysm from the SVC. Any compression of the adjacent structures, thrombus formation, or pulmonary embolism were not evident. First, we performed the coil embolization of the caudal AVA inflow and the second, third, and fourth intercostal veins via the right internal jugular vein approach (Fig. 2). Next, through a reversed L-shaped sternotomy (right fourth intercostal space) (Fig. 3A), a cardiopulmonary bypass was established by right femoral artery cannulation and venous drainage from the right atrium and both brachiocephalic veins. Intraoperative findings revealed no adhesion of the AVA to the lung and SVC (Fig. 3B); therefore, we considered AVA resection. The orifice of the azygos vein was 20 × 50 mm on the posterior wall of the SVC (Fig. 3C). Subsequently, AVA was depressurized, allowing the aneurysm excision by a stapler. We excised the AVA with a stapler device (Powered ECHELON FLEX® 7; Ethicon, Tokyo, Japan) and closed the orifice. (Fig. 3D) The cardiopulmonary bypass time was 64 min, operative time was 394 min, and bleeding volume was 105 ml. Histopathologic examination confirmed the preoperative diagnosis; it showed few inflammatory cell infiltration around vasa vasorum, intimal thickening and hypertrophy of the medial smooth muscle cell. The subsequent surgical and clinical courses were uneventful, and the patient was discharged in stable condition 8 days later. Postoperative CT showed no abnormalities, with the aneurysm having disappeared.

## Discussion

The main causes of AVAs are idiopathic, acquired, and traumatic. Based on the form and medical history of our case, we believe that it was an idiopathic saccular aneurysm that was congenital. In patients with AVA, there are risks of rupture, thromboembolism, and symptoms caused by compression of adjacent structures [1, 4]. It was reported that intra-aneurysmal turbulent blood flow through the saccular AVA may increase the risk of thrombus formation, causing progressive enlargement of the AVA. Therefore, saccular AVAs may have a higher frequency of AVA enlargement and intraluminal thrombosis than fusiform AVAs [1, 4]. In our case, although thrombosis was not evident, the aneurysm was excised to avoid the risks of future rupture and thromboembolism. Video-assisted thoracic surgery (VATS) has been widely performed for small AVAs. When the AVA is very large or adhesions around the AVA are observed, open thoracotomy is reported to be safer than VATS, which has the risk of massive haemorrhage and intraoperative thrombus migration [4, 6]. We believed that this case was too large to be resected using VATS and, hence, performed a surgical resection through a reversed L-shaped sternotomy. However, based on the surgical findings, it was thought that the lesion could have been ligated the AVA even with a small open thoracotomy from a retrospective point of view. As a future issue, we believe that endoscopic observation of the pleural cavity would have allowed us to determine the degree of adhesion and to select a less invasive technique at the start of surgery. In addition, if the size of the AVA was small and the morphology of the aneurysm was resectable, even off-pump resection would be feasible. Open thoracotomy with coil embolization reduces the risk of massive haemorrhage during and is easy to perform because of the depressurisation of the AVA. This sternotomy did not limit the surgical field.

## Conclusion

Reversed L-shaped thoracotomy is a good approach for surgically treating AVA owing to excellent visualisation.

## Data Availability

None.

## Abbreviations

AVA: Azygos vein aneurysm
CT: Computed tomography
MRI: Magnetic resonance imaging
IVR: Interventional radiology
VATS: Video assisted thoracic surgery
SVC: Superior vena cava
